# Body density of humpback whales (*Megaptera novaengliae*) in feeding aggregations estimated from hydrodynamic gliding performance

**DOI:** 10.1371/journal.pone.0200287

**Published:** 2018-07-12

**Authors:** Tomoko Narazaki, Saana Isojunno, Douglas P. Nowacek, Rene Swift, Ari S. Friedlaender, Christian Ramp, Sophie Smout, Kagari Aoki, Volker B. Deecke, Katsufumi Sato, Patrick J. O. Miller

**Affiliations:** 1 Sea Mammal Research Unit, University of St Andrews, Fife, United Kingdom; 2 Atmosphere and Ocean Research Institute, University of Tokyo, Kashiwa, Chiba, Japan; 3 Nicholas School of the Environment and Pratt School of Engineering, Duke University Marine Laboratory, Beaufort, North Carolina, United States of America; 4 Institute of Marine Sciences, University of California Santa Cruz, Santa Cruz, California, United States of America; 5 Mingan Island Cetacean Study, Longue-Pointe-de-Mingan, Québec, Canada; 6 Department of Science, Natural Resources and Outdoor Studies, University of Cumbria, Ambleside, United Kingdom; Sanya Institute of Deep-sea Science and Engineering Chinese Academy of Sciences, CHINA

## Abstract

Many baleen whales undertake annual fasting and feeding cycles, resulting in substantial changes in their body condition, an important factor affecting fitness. As a measure of lipid-store body condition, tissue density of a few deep diving marine mammals has been estimated using a hydrodynamic glide model of drag and buoyancy forces. Here, we applied the method to shallow-diving humpback whales (*Megaptera novaeangliae*) in North Atlantic and Antarctic feeding aggregations. High-resolution 3-axis acceleration, depth and speed data were collected from 24 whales. Measured values of acceleration during 5 s glides were fitted to a hydrodynamic glide model to estimate unknown parameters (tissue density, drag term and diving gas volume) in a Bayesian framework. Estimated species-average tissue density (1031.6 ± 2.1 kg m^-3^, ±95% credible interval) indicates that humpback whale tissue is typically negatively buoyant although there was a large inter-individual variation ranging from 1025.2 to 1043.1 kg m^-3^. The precision of the individual estimates was substantially finer than the variation across different individual whales, demonstrating a progressive decrease in tissue density throughout the feeding season and comparably high lipid-store in pregnant females. The drag term (*C*_*D*_*Am*^-1^) was estimated to be relatively high, indicating a large effect of lift-related induced drag for humpback whales. Our results show that tissue density of shallow diving baleen whales can be estimated using the hydrodynamic gliding model, although cross-validation with other techniques is an essential next step. This method for estimating body condition is likely to be broadly applicable across a range of aquatic animals and environments.

## Introduction

The body condition of animals influences survival rate and reproductive success and thereby impacts the dynamics of entire populations. Body condition also affects an animal’s behavioural decisions related to foraging, predator avoidance, migration, and reproductive strategies (e.g. [1–3]). Many marine mammals undergo substantial changes in lipid-store body condition as a result of annual fasting and feeding cycles [4, 5]. For migratory species, the cost of reproduction at breeding grounds is supported by energy gained on feeding grounds. Thus, the amount of energy stored during a feeding season strongly influences reproduction via pregnancy rate [6], foetal development [7], body condition and survival of offspring [8–11] and the competitive capabilities of males. It is also likely that body condition influences the foraging decisions made by baleen whales relative to where prey items are located in the water column [12, 13]. Because body condition is an important factor affecting fitness, measuring body condition of free-ranging cetaceans is essential for understanding their ecology as well as for designing effective conservation plans [14–16].

Baleen whales (parvorder Mysticeti; order Cetartiodactyla) are a group of marine mammals that cycle fat stores on an annual basis, substantially changing their appearance, behaviour, and fitness [17]. Given these dramatic changes, developing methods to quantify their body condition in the field has great value. Traditional approaches to examine variations in body condition and energy store of baleen whales involved anatomical measurements that were often made in conjunction with whaling operations [4, 18, 19]. Blubber thickness of whale carcasses has been used as a proxy of body condition [4, 18, 20], since most of the energy is stored in the form of blubber [21] although a considerable amount of energy is also stored in muscle and intra-abdominal fat [4, 21]. Blubber lipid content of whale carcasses was also important in the assessment of condition of cetaceans [19]. As the thickness as well as lipid content and fatty acid composition of blubber has been shown to vary across the body of cetaceans, multiple-site measurements of blubber thickness are particularly useful to examine total body condition of cetaceans [22–24]. Many studies have investigated seasonal trends in energy storage of several species of baleen whales by means of blubber thickness and morphometric data, reporting that seasonal fattening varies with different sex and age classes, reproductive stages, as well as prey availability [4, 6, 25].

Although carcasses have provided many insights into the physiology and body condition of baleen whales, a key limitation is that temporal changes of the same individual cannot be measured. Also, studies using carcasses may not be widely applicable to cetaceans because they require lethal sampling or collection of samples from stranded animals or fisheries bycatch. To collect blubber and other tissue samples from free-ranging cetaceans, biopsy darting is commonly used where modified dart tips are delivered using a crossbow or a pneumatic rifle [26]. The percentage lipid content of blubber from carcasses is considered to be an informative measure of fattening [19]. However, the biopsy blubber samples may not be useful to measure body condition of free-ranging cetaceans because (1) the force of darting can damage adipocytes causing lipids to be squeezed out of samples, or to seep out of blubber biopsies while in seawater [27], and (2) the sample only penetrates a short distance into the blubber layer. In addition, it is difficult to obtain multiple biopsy samples from a whale whose blubber thickness and composition vary across the body [22–24]. Visual assessment of external shape and appearance based on boat-based photographs has been used for evaluating body condition of right and grey whales [28, 29]. Photogrammetric measurements of body width, reflecting blubber thickness, using vertical aerial photographs taken from aircraft or unmanned aerial vehicles has also been used to assess nutritive body condition of some whale species [11, 30, 31] although measurements of such body shape patterns are limited to the visible 2-dimensionall shape of surfacing of whales, and may not be suitable for other more cryptic species.

An alternative approach is to use body density of diving animals as a proxy of lipid-store body condition [32]. Lipids are less dense than seawater while other non-gas body components are denser than seawater. Body composition, particularly the ratio of lipid to lean tissue, therefore strongly influences body density and hence the buoyancy of diving animals [5]. It has been shown that buoyancy influences swimming behaviour and energetics of diving animals [33]. For instance, buoyancy forces affect stroking efforts [34, 35] and swimming patterns, with more gliding occurring in the direction aided by buoyancy [15, 34, 36–39].

Buoyancy also influences gliding performance by altering vertical speeds during inactive drift periods [5], prolonged glides [39] or short-duration glides [38]. This effect of buoyancy on gliding performance has led to the development of tag-based methods to quantify the body density of diving animals via hydrodynamic analysis. This approach was first developed for free-ranging elephant seals (*Mirounga* spp.): body density was quantified by analysing the vertical speed during inactive drifting periods (i.e. drift rate) at which the buoyancy force is assumed to be equal to the drag force [5]. The drift dive method has proven useful for long-term monitoring of body lipid-stores in elephant seals providing new insights into when and where they gain or lose lipid stores [5, 40, 41]. However, use of the drift dive method is limited to a few pinniped species that routinely perform drift dives (*Mirounga angustirostris* [34, 42]; *M*. *leonina* [5, 40]; *Arctocephalus forsteri* [43]; *Cystophora cristata* [44]). Gliding during the descent or ascent phase of a dive, on the other hand, is commonly observed across a range of diving taxa [33, 37]. A more widely applicable approach, the glide model, was introduced by Miller et al. [38] to estimate body density of sperm whales using a hydrodynamic glide model that predicts how drag and buoyancy forces influence acceleration (or deceleration) during short-duration glides. Aoki et al. [34] conducted a validation analysis using isotope dilution and confirmed a strong correspondence in body density estimates of elephant seals obtained from the drift dive and the glide models [45].

In the glide model, acceleration during a glide is determined by the difference between drag and net buoyancy forces along the swimming path of the animal [38]. The force of non-neutral buoyancy or ‘apparent weight’ (difference in mass of the diving animal and the displaced water) acts vertically on diving animals, and depends on the density of body tissues as well as the volume of air carried within the body (the diving gas volume). While body tissues are relatively incompressible at depth, the volume of air in the body progressively decreases with increasing depth, thought to closely follow Boyle’s Law for marine mammals [46]. Thus, tissue-derived buoyancy can be separated from air-derived buoyancy when gliding data is available over a wide depth range. To date, the glide model has been demonstrated to be useful to estimate the body density of several species of marine mammals, including elephant seals [34] and some deep diving toothed whales (sperm whale, *Physeter macrocephalus* [38]; Northern bottlenose whale, *Hyperoodon ampullatus* [15]; long-finned pilot whale, *Globicephala melas* [47]) that routinely perform dives deeper than 200 m where the effect of air-derived buoyancy is considered to be negligible [38].

In this study, we apply the hydrodynamic glide model to estimate body tissue density of humpback whales (*Megaptera novaeangliae*) in two geographically distinct feeding populations (the Gulf of St Lawrence, Canada and the Western Antarctic Peninsula, WAP). In comparison with deeper diving toothed whales, humpback whales may not seem ideal candidates for the glide model because they routinely dive only to relatively shallow depths at which gas volumes are likely to more strongly influence net buoyancy. For example, the mean dive depth per tag record in this study ranged from 22.8 to 180.8 m, with the deepest dive recorded being 388.3 m. Apart from a shallower diving depth range, humpback whales tend to dive and glide at relatively shallower pitch angles, requiring the generation of lift. The large flippers of humpback whales are well-suited for this purpose [48], but the need to generate substantial lift forces may raise concerns about the applicability of the glide model because the current model does not include the potential effect of lift-induced drag which was shown to be negligible in deep divers that maintain steep pitch during glides [34].

The objective of this study was to examine whether the hydrodynamic glide model can be applied to shallower diving baleen whales by examining the precision of body density estimates obtained from a narrow depth-range dataset. Our results show that we were able to obtain estimates of humpback whale body density using this method. Though the precision of the estimates was not as fine as was previously reported for a deep-diving toothed whale [15], the precision of individual body density estimates was substantially finer than the variation across different individual whales, including some differences between the geographic locations where tags were attached. We conclude that the glide method has potential to be used to track the body condition of shallow diving baleen whales, enabling future applications as a tool to study their health and how body condition relates to reproductive status, animal behaviour and the influences of environmental change and variability.

## Materials and methods

### Ethics statement

The research protocol was approved by Animal Welfare and Care Committee Approval of the University of St Andrews. The fieldwork in the Gulf of St Lawrence, Canada was performed under the Research permits issued by Department of Fisheries and Oceans, Canada (scientific fishing license QUE04-B-2011) in compliance with ethical and local use of animals in experimentation. All research activities in the Antarctic was conducted under National Marine Fisheries Service Permit (808–1735), Antarctic Conservation Act Permit (2009–014), and Duke University Institutional Animal Care and Use Committee (A049-112-02).

### Data collection

Field studies were carried out at two geographically distinct summer feeding grounds of humpback whales (*Megaptera novaeangliae*): the Gulf of St Lawrence in Canada (49.7–50.0N, 63.4–65.0W) and the western side of Antarctic Peninsula (64.4–64.9S, 61.8–63.1W). Animal-borne archival tags used in the study were either 3MPD3GT loggers (Little Leonardo Co., Tokyo, Japan) or sound and movement recording DTAGs ([49]; Table 1). The 3MPD3GT loggers were programmed to record depth, temperature, flywheel swim speed and 3-axis magnetism at 1Hz, and 3-axis ± 3 *g* acceleration at 32Hz. The DTAG sampled pressure and a 3-axis ± 2 *g* acceleration at 50Hz, which was later downsampled to 5Hz. The 3MPD3GT loggers have the ability to measure flow speed using a front mounted impeller (flywheel). To ensure that speed is measured in the direction of travel, 3MPD3GT tags are mounted in hydrodynamic (tear shaped) floats with a single suction cup mounted at the anterior end, and vertically mounted tail fin at the posterior end. The location of fin and suction cup ensure that the force acting on the tag cause the tag housing to swivel on the animal and orient into the direction of flow. DTAGs are attached to the animal with four suction cups. Tagging was conducted from rigid-hull inflatable boats and either a 5 m or an 8 m handheld carbon fibre pole was used to attach the tag.

**Table 1:** Humpback whale dataset used for analysis.

Data ID	Date	Location	Duration (h)	Tag type	Age class	Sex	No of 5-s glides	*ρ*_*tissue*_ (kg m^-1^)	*C*_*D*_*Am*^*-1*^ (x10^-6^ m^2^ kg^-1^)
Mn11_H584_1	21 Jul 2011	GSL	0.2	3MPD3GT	Adult (pregnant)	F	0	N/A	N/A
Mn11_H607_1	22 Jul 2011	GSL	3.4	3MPD3GT	Adult	M	23	1037.0 ± 1.9	12.5 ± 1.4
Mn11_H686	25 Jul 2011	GSL	4.5	3MPD3GT	Adult	F	75	1036.2 ± 1.2	6.5 ± 2.1
Mn11_H761	25 Jul 2011	GSL	5.9	3MPD3GT	Adult	M	44	1029.0 ± 1.8	16.3 ± 6.5
Mn11_H731	26 Jul 2011	GSL	2.7	3MPD3GT	Adult	F	61	1035.4 ± 1.2	12.8 ± 2.0
Mn11_H698	26 Jul 2011	GSL	2	3MPD3GT	Adult	M	61	N/A	N/A
Mn11_H228	27 Jul 2011	GSL	0.2	3MPD3GT	Adult	F	3	N/A	N/A
Mn11_H584_2	28 Jul 2011	GSL	3.6	3MPD3GT	Adult (pregnant)	F	47	1028.6 ± 0.7	12.2 ± 1.4
Mn11_H707	19 Aug 2011	GSL	1.6	3MPD3GT	Juvenile	M	93	1043.1 ± 1.6	12.4 ± 1.5
Mn11_H755	28 Aug 2011	GSL	2.9	3MPD3GT	Juvenile	M	177	1033.7 ± 0.5	25.5 ± 1.0
Mn11_H607_2	01 Sep 2011	GSL	2.1	3MPD3GT	Adult	M	29	1031.2 ± 2.3	15.0 ± 9.6
Mn11_H002	04 Sep 2011	GSL	5.8	3MPD3GT	Adult (pregnant)	F	187	1026.5 ± 0.5	6.3 ± 2.8
Mn11_H405	18 Sep 2011	GSL	2.7	3MPD3GT	Adult	M	74	1034.2 ± 0.9	13.1 ± 1.1
Mn11_H489	19 Sep 2011	GSL	0.1	3MPD3GT	Adult	F	0	N/A	N/A
Mn09_121	01 May 2009	A	6.4	Dtag	Adult	U	7	N/A	N/A
Mn09_122	02 May 2009	A	4.2	Dtag	Adult	U	5	N/A	N/A
Mn09_127a	07 May 2009	A	24.2	Dtag	Adult	U	290	1028.4 ± 0.1	11.7 ± 0.2
Mn09_127b	07 May 2009	A	6.5	Dtag	Adult	U	15	N/A	N/A
Mn09_128	08 May 2009	A	2.4	Dtag	Adult	U	11	N/A	N/A
Mn09_136	16 May 2009	A	22.5	Dtag	Adult	U	704	1028.7 ± 0.03	11.2 ± 0.2
Mn09_140	20 May 2009	A	22.3	Dtag	Adult	U	500	1029.8 ± 0.04	9.8 ± 0.2
Mn09_148	28 May 2009	A	25.5	Dtag	Adult	U	30	1026.9 ± 0.7	10.1 ± 2.1
Mn09_151	29 May 2009	A	3.1	Dtag	Juvenile	F	5	N/A	N/A
Mn09_152	01 Jun 2009	A	22.4	Dtag	Adult	U	230	1036.3 ± 0.4	10.5 ± 1.3
Mn10_133	13 May 2010	A	22.8	Dtag	Adult	F	86	1028.6 ± 0.3	6.6 ± 2.4
Mn10_139a	19 May 2010	A	22.2	Dtag	Calf of Mn10_139b	F	118	1040.8 ± 0.5	14.7 ± 0.9
Mn10_139b	19 May 2010	A	23.7	Dtag	Adult	F	457	1029.4 ± 0.1	17.4 ± 1.5
Mn10_143	23 May 2010	A	23.3	Dtag	Unknown	U	77	1026.4 ± 0.3	22.3 ± 3.1
Mn10_144	24 May 2010	A	19.9	Dtag	Adult	M	47	1031.1 ± 0.9	6.0 ± 3.5
Mn10_146	26 May 2010	A	20.2	Dtag	Adult	F	419	1029.7 ± 0.1	11.6 ± 0.1
Mn10_151	31 May 2010	A	25	Dtag	Juvenile	F	352	1035.3 ± 0.2	14.0 ± 0.5
Mn10_155a	04 Jun 2010	A	24.2	Dtag	Adult	F	391	1027.6 ± 0.1	14.1 ± 0.3
Mn10_155b	04 Jun 2010	A	22	Dtag	Calf of Mn10_155a	F	67	1025.2 ± 0.4	12.8 ± 1.9

GSL and A in the Location column indicate Gulf of St. Lawrence and Antarctica, respectively. Individual-specific estimates of tissue density (*ρ*_*tissue*_) and the combined drag term (*C*_*D*_*Am*^*-1*^) obtained from the lowest DIC model are presented as mean ± 95% credible interval. Data was not used for the Bayesian estimation when number of 5-s glides was < 20. Dataset shaded with grey were not used for the Bayesian estimation due to insufficient number of 5-s glides in the dataset.

### Analysis of tag data

Pressure data recorded by archival tags were converted to absolute values of hydrostatic pressure using calibration values and converted to meters. A dive was defined as any submergence to a depth of > 10 m. Dives were broken into descent, bottom and ascent phases based on changes in pitch following Miller *et al*. [38]. As tags were attached to whales at random orientations, the 3-axis acceleration data recorded by the tags was converted to a whale-centred, whale fixed reference frame (whale-frame) using established methods [38, 49]. The accelerometers recorded both specific (e.g. stroking) and gravity-based accelerations (i.e. changes in response to posture change). Under the assumption that changes in the posture of the tagged whale occurred at lower frequency than changes in accelerations resulting from body motions such as thrust, a frequency-based filter (low-pass finite impulse response filters with tag-specific thresholds set at 0.12–0.15 Hz) was applied to the entire acceleration time-series to separate these two components. Then, pitch and roll angles of the whales were calculated from the low-frequency component of accelerations [37, 39, 50], while the high-frequency component was used to identify stroking versus gliding periods. For 3MPD3GT dataset, stroking was identified when oscillation on the high-frequency component of surge accelerations indicating fluke beats exceeded a threshold that was set for each deployment (0.1–0.2 m s^-2^). Speed sensor data was visually inspected to confirm the presence of stroke-derived acceleration. For DTAG dataset, stroking was detected using high-frequency accelerations at both surge and dorso-ventral axis with thresholds set for each deployment and each axis (0.1–0.2 m s^-2^). Gliding periods were automatically detected as the period when the tagged animals did not stroke.

The speed sensor of the 3MPD3GT logger recorded swim speed as the rotation of an external impeller mounted on the anterior end of the logger, which correlates linearly with the speed of water flow passing through the impeller. The rotation rate (number of rotations per second) was converted to speed (m s^-1^) using a calibration line obtained in-situ for each deployment [37]. The calibration line was obtained from a linear regression of rotation rate against swim speed that was calculated from vertical depth change divided by sine of the pitch at 5 s intervals when absolute mean sine of pitch was greater than 0.7–0.9. For the DTAG data, speed during glides was estimated using the rate of change of depth divided by the sine of pitch [38].

Data during glides were extracted in 5 s duration segments [15]. Glides shorter than 5 s were excluded from the analysis and glides longer than 5 s were broken into 5 s sub-glides. For each 5 s sub-glide, mean depth (*d*), speed (*v*) and pitch angle (*p*) were calculated. Acceleration (*a*) was measured by regressing speed versus time over each 5-second interval (S1 Fig). The variance of the acceleration measurement during each 5 s sub-glide was quantified as the root mean square of residuals from the fitted regression line. Seawater density (*ρ*_sw_) for each sub-glide was calculated from a CTD cast that was made close in time and location to each tagged whale. In this analysis, we only used stable glides (circular variance of roll < 0.1) that were at steep pitch angle (absolute pitch > 30°) to enable robust estimates of speed for DTAG records. In addition, any glides associated with lunge feeding were excluded from the analysis because body form and kinematics of whales drastically change during this feeding behaviour [51]. Lunge feeding events were detected as peaks in jerk (i.e. differential of acceleration) for DTAG records [52]. For 3MPD3GT records with speed data, a lunge was detected as peak in speed when the speed exceeded the threshold of mean speed plus two standard deviations followed by a rapid deceleration. According to a fine-scale kinematic study of lunge-feeding humpback whales, whales stroke throughout lunges but glide at the end of feeding once the mouth has been closed [52]. To exclude any feeding-related glides, we excluded any glides recorded within 46 s after the lunge from the analysis because it has been reported that humpback whales spend an average of 46 s for filtration and prey handling [52].

### Hydrodynamic performance model

We used the equation presented by Miller *et al*. [15] where acceleration (m s^-2^) along the swimming path is determined by drag force (the first term) and buoyancy forces derived from body tissue (the second term) and gasses carried by each whale (the third term):

$$\mathrm{Acceleration}=-0.5∙\frac{C_{D}∙A}{m}∙\rho_{sw}∙v^{2}+\left(\frac{\rho_{sw}}{\rho_{tissue}(d)}-1\right)∙g∙\sin\left(p\right)+\frac{V_{air}}{m}∙g∙\sin\left(p\right)∙\frac{\rho_{sw}-\rho_{air}∙(1+0.1∙d)}{\left(1+0.1∙d\right)}$$

where:

$$\rho_{tissue}(d)=\frac{\rho_{tissue}(0)}{1-r∙(1+0.1∙d)∙101325∙10^{-9}}$$

Here, *C*_*D*_ is the drag coefficient, *A* is the relevant surface area (m^2^), *m* is the mass of the whale (kg), *ρ*_*sw*_ is the density of the surrounding seawater (kg m^-3^), *v* is swim speed (m s^-1^), *ρ*_*tissue*_ is the density of the non-gas component of the whale body (kg m^-3^), *g* is acceleration due to gravity (9.8 m s^-2^), *p* is animal pitch (radians), *V*_*air*_ is the volume of air at the surface (m^3^), *ρ*_*air*_ is the density of air (kg m^-3^), *d* is glide depth (m), and *r* is compressibility for animal tissue (i.e., the fractional change in volume per unit increase in pressure). The value 101325 converts pressure in atmospheres to pressure in Pascals, so that the units of body tissue compressibility are proportion per Pascal x 10^−9^.

The first additive term of the equation represents the effect of drag on the forward motion of the whale during a glide, which is primarily a function of speed itself. *C*_*D*_*Am*^-1^is the unknown term that is treated as a single quantity in this approach with units of m^2^ kg^-1^. The second term quantifies the effect of net buoyancy derived from unknown tissue density (*ρ*_*sw*_) on speed during a glide. The third term quantifies the influence of net buoyancy derived from the unknown volume of gas per unit mass carried in the dive (*V*_*air*_*m*^-1^) on speed during a glide. As gas compartments of whales are compressed during dives, the volume and density of gas carried by the animal are modelled to change with hydrostatic pressure following Boyle’s Law. The model also includes the effect of tissue compressibility (*r*) that was fixed as 0.38 x 10^−9^ Pa^-1^ based on the value estimated for northern bottlenose whales [15].

### Bayesian estimation

The unknown parameters in the hydrodynamic glide model (mainly *ρ*_*tissue*_, *V*_*air*_*m*^-1^ and *C*_*D*_*Am*^-1^) were estimated by Bayesian Gibbs sampling with the freely available software JAGS within R (coda, R package v0.17–1 2015, http://cran.r.project.org/web/packages/coda/index.html) and R2jags (R package v0.5–7 2012, https://cran.r-project.org/web/packages/R2jags/index.html) using data extracted for each 5-s sub-glide. Acceleration during glides was measured using a linear regression line of speed versus time. Observation error measured from variance of acceleration for each 5 s was incorporated in the model by treating acceleration as a normal variable with a precision parameter (1/variance) [15]. A small increment (0.001) was added to the standard errors to ensure finite values for the precision parameter. For the Bayesian estimation, a specific prior distribution must be set for each unknown parameter. A non-informative uniform prior from 800 to 1200 kg m^-3^ was set for body tissue density (*ρ*_*tissue*_). An informative prior was set for the combined drag coefficient term (*C*_*D*_*Am*^-1^) based on several sources of information: drag coefficient (*C*_*D*_) was estimated to be 0.0026 based on the value estimated for a fin whale (*Balaenoptera physalus*) swimming at 4 m s^-1^ [53]. Based on body lengths (*L*) ranges from 6 to 15 m, body mass (*m*) was estimated as 20005 kg on average (range 3253–48556 kg) using an equation derived for humpback whales: *m* = 0.016473*L*^2.95^ x 1000 [54]. Surface area (*A*) was estimated as 47.4 m^2^ (range 15.3–89.0 m^2^) using a prediction equation obtained from bottlenose dolphins (*Tursiops truncatus*): A = 0.08*m*^0.65^ [55]. Thus, an expected value for the combined drag term (*C*_*D*_*Am*^-1^) would be 7 x 10^−6^ m^2^ kg^-1^, with a range from 5 x 10^−6^ m^2^ kg^-1^ for large whales to 12 x 10^−6^ m^2^ kg^-1^ for small whales. In order to capture uncertainty around this expected value, we specify the prior to be a normal distribution with a mean of 7 x 10^−6^ m^2^ kg^-1^ and standard deviation of 2 x 10^−6^ m^2^ kg^-1^ that was truncated at 1 x 10^−6^ m^2^ kg^-1^ and 20 x 10^−6^ m^2^ kg^-1^. For diving gas volume (*V*_*air*_*m*^-1^), a uniform prior from 5 to 80 ml kg^-1^ was set based on the total lung capacity (65–72 ml kg^-1^) estimated for 6 to 15 m long whales using an equation derived from various marine mammals: total lung capacity = 0.10*m*^0.96^ x 1000 [56].

Following Miller et al. [15], we explored variability of unknown tissue density, combined drag term and diving gas volume by evaluating a total of 12 model structures. We fitted a model in which the quantity of the unknown parameters *ρ*_*tissue*_, *V*_*air*_*m*^-1^ and *C*_*D*_*Am*^-1^ remained constant across the tags and dives (global estimates). We also fitted hierarchical models in which the individual-specific estimates of tissue density and/or drag term, and the dive-specific estimates for diving gas volume are sampled from each global (i.e. individual-average or dive-average) distribution that was estimated for each parameter. See the JAGS script in the appendix of Miller et al. [15] for the detailed structure of the hierarchical model. All models were sampled in three independent chains, with 24,000 iterations each. The first 12,000 samples were discarded for burn-in, and the remaining posterior samples were downsampled by a factor of 36 to remove any serial correlation in the samples. We report the mean and 95% percentile, hereafter termed posterior mean and credible interval (CI), of the posterior samples as the best estimates of the parameter value and its uncertainty. The 95% credible interval is the Bayesian analogue for the more traditional (frequentist) confidence interval, and defines the range of values within which the true parameter value lies with 95% probability, given the observed data. Convergence was assessed for each parameter, using trace history and Brooks-Gelman-Rubin diagnostic plots [57]. The best model was selected based on the deviance information criterion (DIC), with a lower value indicating a better model fit relative to model complexity.

## Results

**Fig 1:**
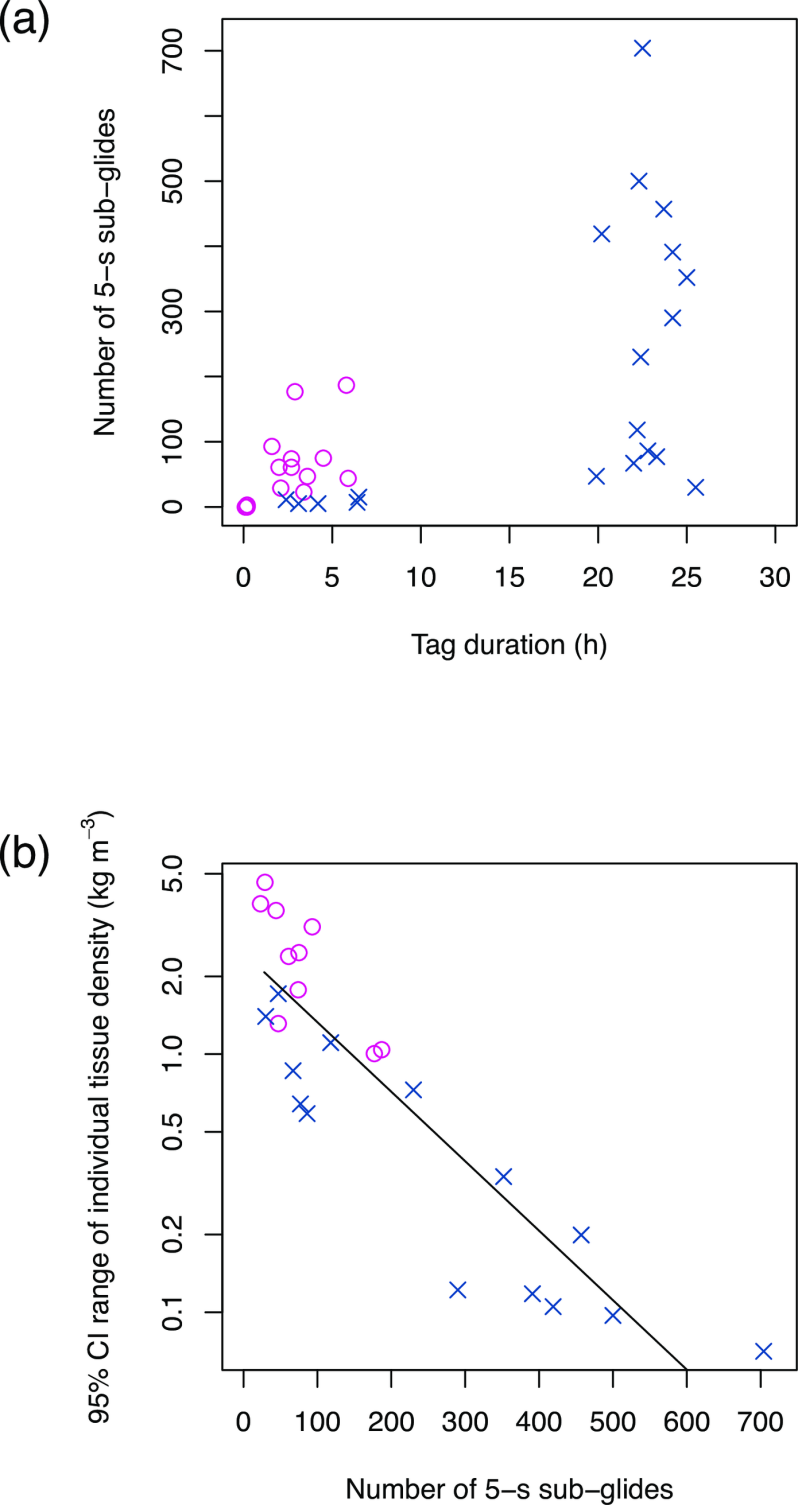
The number of 5-s sub-glides in relation to tag duration and 95% CI range. Number of 5-s sub-glides that could be used for the hydrodynamic glide model in relation to tag duration (a) and the range of 95% credible interval for tissue density estimates (b). Magenta circles and blue crosses indicate data from Gulf of St Lawrence and Antarctica, respectively. A solid line shows a regression line: log(y) = -0.0062x + 0.90.

A total of 33 tag datasets were analysed (Table 1). In the Gulf of St Lawrence, archival tags were deployed on 12 whales in the Jacques-Cartier Passage and adjacent waters between July and September 2011. All tagged whales were part of a long-term photo-identification study that has been carried out at the study site since 1984 [58]. Photographic and field observations of behaviour and known associates suggest that at least two adult females (H002 and H584_2) were pregnant when the tag data were collected. Pregnancy of H002 was also confirmed by hormonal analysis of blow samples and blubber samples. One adult male (H607) was tagged twice at the beginning of the feeding season (July 22, 2011) and later the same season (September 1, 2011). At the Antarctic field-site, 19 whales were tagged over the course of two field seasons that ran between May and June in both 2009 and 2010. Antarctic animals were tagged in Wilhelmina and Andvord Bays along the WAP and inshore waters of the Gerlache Strait. Two pairs of tagged whales were found to be mother-and-calf pairs based on visual observation from the tag boat and biopsy samples (Table 1).The whales conducted dives to a maximum depth of 388.3 m. Mean swim speed throughout dives was 1.5 ± 0.4 m s^-1^ (± SD, Table 2). Gliding was observed both during descent and ascent phases although the percentage of time spent gliding varied among whales ranging over 1.5–45.2% and 2.8–60.0% during descent and ascent phases, respectively. Pitch angles during descent and ascent phases were -39.8 ± 20.6° and 30.6 ± 22.4° on average, respectively (Table 2). From the whole dataset, we extracted a total of 18546 5-s sub-glides that were not associated with a lunge. However, 73.7% of these glides were filtered out due to shallow pitch angle (< 30°) and 0.1% due to high variability in roll (circular variance of roll > 0.9). In addition, 1.4% of glides were removed due to lack of speed and/or acceleration data throughout the 5-s glides. As a result, 24.7% of the total 5-s sub-glides met the criteria for the use of hydrodynamic glide model. The number of 5-s sub-glides that could be used for the hydrodynamic glide model was positively correlated with the duration of tag dataset (Spearman’s rho = 0.633, p < 0.001; Fig 1A) although the number of useable glides also varied depending on the behaviour of the tagged whales (foraging, resting, etc.). Eight tag datasets were excluded from the Bayesian estimation of tissue density because of insufficient sample size (<20 sub-glides in each dataset; Table 1). Data of Mn11_H698 was also excluded because an in-situ calibration of the speed sensor was not applicable for this deployment.

**Table 2:** Summary of dive statistics.

	*Dives*				*Descent phase*				*Ascent phase*		
Data ID	N	Duration (s)	Depth (m)	Speed (m s^-1^)	Duration (s)		Pitch (°)	% time gliding	Duration (s)	Pitch (°)	% time gliding
Mn11_H584_1	0	N/A	N/A	N/A	N/A	N/A		N/A	N/A	N/A	N/A
Mn11_H607_1	68	133.1 ± 70.6	45.1 ± 14.4	2.1 ± 0.3	50.8 ± 24.6	-26.0 ± 10.7		7.7 ± 14.4	42.4 ± 20.7	28.8 ± 8.7	6.0 ± 11.3
Mn11_H686	32	204.7 ± 105.8	56.7 ± 60.7	1.3 ± 0.4	63.6 ± 47.7	-18.1 ± 13.5		14.5 ± 21.0	85.8 ± 46.4	23.9 ± 24.5	8.8 ± 16.6
Mn11_H761	51	158.4 ± 110.1	25.3 ± 35.7	1.0 ± 0.3	37.8 ± 26.8	-23.4 ± 14.2		21.1 ± 29.5	91.7 ± 85.6	19.0 ± 13.0	35.1 ± 33.5
Mn11_H731	33	140.9 ± 81.6	44.3 ± 43.6	1.3 ± 0.4	52.5 ± 37.2	-22.0 ± 17.0		31.1 ± 26.9	67.6 ± 41.5	20.8 ± 10.8	38.1 ± 31.8
Mn11_H698	43	101.1 ± 48.3	32.6 ± 18.5	N/A	25.4 ± 8.9	-28.7 ± 16.8		37.3 ± 28.3	56.9 ± 30.1	20.1 ± 9.5	34.1 ± 26.6
Mn11_H228	4	158.0 ± 85.1	24.9 ± 12.2	1.5 ± 0.5	38.5 ± 7.1	-21.7 ± 11.5		42.5 ± 31.4	99.0 ± 80.4	13.0 ± 1.8	60.0 ± 41.4
Mn11_H584_2	32	256.4 ± 153.1	70.0 ± 54.7	2.2 ± 0.6	75.6 ± 41.5	-22.7 ± 16.8		5.5 ± 13.9	96.2 ± 73.9	19.2 ± 9.8	16.1 ± 26.2
Mn11_H707	17	294.1 ± 162.5	116.4 ± 58.6	2.0 ± 0.3	50.1 ± 26.5	-39.2 ± 19.0		45.2 ± 27.5	70.1 ± 30.6	45.7 ± 26.1	6.1 ± 15.4
Mn11_H755	28	328.0 ± 203.0	73.4 ± 45.9	1.3 ± 0.3	58.7 ± 47.1	-32.1 ± 16.4		37.1 ± 33.5	93.4 ± 66.6	19.8 ± 11.8	41.3 ± 31.2
Mn11_H607_2	13	210.0 ± 107.2	64.2 ± 51.3	1.2 ± 0.3	65.2 ± 36.1	-26.2 ± 19.5		19.9 ± 24.3	95.1 ± 79.1	21.0 ± 12.5	8.3 ± 17.8
Mn11_H002	57	217.0 ± 107.2	51.9 ± 41.6	1.3 ± 0.3	63.8 ± 45.3	-18.8 ± 9.3		24.8 ± 24.0	90.4 ± 67.0	15.7 ± 10.4	39.5 ± 31.0
Mn11_H405	24	333.7 ± 194.1	76.6 ± 44.4	1.9 ± 0.3	70.8 ± 36.3	-25.1 ± 12.0		31.9 ± 21.6	72.3 ± 36.9	25.2 ± 14.5	12.0 ± 11.2
Mn11_H489	0	N/A	N/A	N/A	N/A	N/A		N/A	N/A	N/A	N/A
Mn09_121	16	215.4 ± 79.0	48.9 ± 36.4	1.3 ± 0.2	77.0 ± 25.7	-28.9 ± 11.6		23.4 ± 15.4	126.6 ± 56.9	15.1 ± 7.6	58.4 ± 34.1
Mn09_122	48	138.6 ± 73.5	22.8 ± 14.2	1.3 ± 0.5	1.8 ± 27.6	-13.0 ± 11.2		4.7 ± 6.3	67.5 ± 38.6	14.3 ± 7.4	13.4 ± 14.5
Mn09_127a	134	299.1 ± 216.7	127.5 ± 116.7	1.4 ± 0.4	93.8 ± 69.2	-43.1 ± 20.7		9.4 ± 12.6	111.9 ± 86.8	38.5 ± 19.5	23.4 ± 22.9
Mn09_127b	29	211.9 ± 133.6	76.9 ± 71.3	1.8 ± 0.7	61.0 ± 41.5	-35.6 ± 8.7		13.0 ± 15.3	118.6 ± 68.8	9.1 ± 10.8	56.9 ± 27.7
Mn09_128	21	243.9 ± 133.9	32.0 ± 14.1	1.2 ± 0.3	81.1 ± 48.6	-27.2 ± 9.6		13.4 ± 18.2	110.8 ± 71.0	10.3 ± 13.6	29.7 ± 27.3
Mn09_136	101	459.3 ± 136.7	180.8 ± 75.9	1.2 ± 0.2	129.8 ± 51.7	-61.6 ± 15.2		17.0 ± 14.5	159.3 ± 56.3	55.7 ± 20.4	36.8 ± 15.8
Mn09_140	141	357.2 ± 181.2	68.8 ± 72.4	1.4 ± 0.4	89.8 ± 56.9	-23.3 ± 14.1		28.5 ± 20.4	99.2 ± 81.1	20.3 ± 11.6	42.3 ± 25.9
Mn09_148	308	127.4 ± 102.9	40.3 ± 34.9	1.6 ± 0.3	40.2 ± 26.9	-37.1 ± 17.3		5.9 ± 10.7	40.3 ± 31.1	29.7 ± 15.3	14.5 ± 22.5
Mn09_151	18	56.6 ± 29.5	175.9 ± 101.6	1.2 ± 0.6	56.6 ± 29.5	-24.0 ± 7.8		37.0 ± 27.0	97.9 ± 71.8	12.3 ± 10.3	43.5 ± 32.2
Mn09_152	326	118.1 ± 111.5	34.9 ± 30.4	1.3 ± 0.3	39.4 ± 34.1	-48.0 ± 17.6		12.5 ± 19.7	34.6 ± 31.6	29.6 ± 12.7	5.3 ± 12.4
Mn10_133	185	217.0 ± 99.5	76.1 ± 41.7	1.6 ± 0.3	72.2 ± 35.1	-40.0 ± 15.2		1.5 ± 4.6	73.0 ± 42.1	32.9 ± 13.2	10.6 ± 13.6
Mn10_139a	288	170.5 ± 112.3	58.7 ± 38.3	1.5 ± 0.2	36.6 ± 28.4	-53.7 ± 18.1		8.6 ± 17.2	56.1 ± 47.7	44.2 ± 15.0	8.5 ± 15.0
Mn10_139b	285	172.1 ± 100.3	45.8 ± 39.3	1.2 ± 0.3	58.6 ± 35.0	-33.0 ± 15.0		7.0 ± 13.5	70.5 ± 53.2	25.5 ± 14.5	22.3 ± 27.1
Mn10_143	286	173.1 ± 107.2	49.5 ± 43.8	1.4 ± 0.5	-54.3 ± 30.1	-32.6 ± 15.3		2.9 ± 7.1	62.4 ± 47.3	25.7 ± 13.6	11.7 ± 18.2
Mn10_144	342	140.8 ± 70.9	52.5 ± 26.1	1.6 ± 0.3	33.2 ± 14.9	-55.9 ± 19.3		4.7 ± 8.6	41.3 ± 24.2	41.0 ± 14.6	2.8 ± 9.1
Mn10_146	88	469.5 ± 147.6	192.5 ± 102.8	1.5 ± 0.3	119.1 ± 47.6	-56.6 ± 22.6		28.4 ± 9.7	143.0 ± 60.8	45.1 ± 26.1	43.6 ± 18.1
Mn10_151	265	191.1 ± 103.7	56.8 ± 41.2	1.4 ± 0.4	53.2 ± 26.6	-44.2 ± 22.4		37.9 ± 31.6	68.8 ± 38.0	29.8 ± 17.7	36.5 ± 33.1
Mn10_155a	153	288.1 ± 179.9	119.3 ± 122.1	1.4 ± 0.4	96.1 ± 73.3	-44.0 ± 21.1		7.9 ± 9.8	96.4 ± 69.6	29.3 ± 27.6	32.6 ± 26.3
Mn10_155b	246	184.2 ± 151.5	58.0 ± 77.1	1.6 ± 0.5	63.9 ± 64.3	-28.5 ± 10.8		5.0 ± 9.3	46.5 ± 43.8	23.1 ± 13.6	8.1 ± 12.4
*ALL*	*3682*	*199*.*7 ± 150*.*1*	*64*.*0 ± 67*.*2*	*1*.*5 ± 0*.*4*	*58*.*6 ± 46*.*8*	*-39*.*8 ± 20*.*6*		*12*.*6 ± 20*.*0*	*68*.*1 ± 57*.*8*	*30*.*6 ± 22*.*4*	*18*.*8 ± 25*.*1*

Mean ± standard deviation were shown. Dataset shaded with grey were not used for the Bayesian estimation due to insufficient number of 5-s glides in the dataset.

Twenty-four of the 33 tag datasets (10 from the Gulf of St Lawrence and 14 from Antarctica) were used to estimate tissue density and the other unknown parameters. Of the 12 Bayesian models, the model with the lowest DIC indicated global plus individual variation in tissue density and drag terms, and global plus dive-by-dive variability in diving lung volume (Table 3). The difference in DIC from the next-best model was 1657.5 units.

**Table 3:** Model parameter values.

*Model fit*	*Model structure*			*Global parameter estimates*					
DIC	*ρ*_*tissue*_	*C*_*D*_*Am*^-1^	*V*_*air*_	*ρ*_*tissue*_.g	*ρ*_*tissue*_.var	*C*_*D*_*Am*^-1^.g	*C*_*D*_*Am*^-1^.var	*V*_*air*_.g	*V*_*air*_.var
28301.1	I	I	D	1031.6 (2.1)	26.5 (17.2)	11.8 (1.6)	23.2 (16.4)	27.7 (1.1)	236.5 (33.4)
29958.6	I	G	D	1031.3 (2.1)	25.4 (16.2)	11.6 (0.1)		26.5 (1.1)	199.8 (25.6)
53399.8	G	I	D	1029.4 (0.02)		7.9 (3.2)	349.8 (369.6)	21.0 (1.4)	353.2 (57.2)
86274.2	G	G	D	1029.7 (0.02)		8.3 (0.1)		20.7 (1.5)	502.8 (89.2)
106380.0	I	I	I	1030.3 (1.8)	19.8 (11.8)	8.5 (2.5)	103.8 (86.0)	25.7 (7.2)	305.2 (288.2)
113957.5	I	G	I	1030.1(1.7)	17.3 (11.1)	9.3 (0.1)		23.5 (5.8)	193.1 (170.0)
120768.9	I	I	G	1029.0 (1.1)	6.5 (4.1)	6.7 (1.8)	39.7 (26.7)	15.6 (0.1)	
125832.6	G	I	I	1029.0 (0.02)		7.3 (3.2)	215.5 (240.8)	19.3 (6.5)	227.8 (238.8)
130603.2	I	G	G	1029.2 (1.13)	7.4 (4.5)	7.9 (0.1)		15.9 (0.1)	
159607.6	G	G	I	1029.2 (0.01)		6.9 (0.1)		24.5 (17.0)	1515.3 (2882.9)
264515.3	G	I	G	1028.1 (0.01)		6.7 (3.5)	1930.7 (7548.2)	8.6 (0.1)	
309390.4	G	G	G	1028.0 (0.01)		1.3 (0.04)		7.2 (0.1)	

Model structure refers to the allowed variation in the model for the unknown terms, with G referring to global (i.e. individual-average) parameter only, I referring to individual specific estimates included, and D referring to dive-by-dive variation included. The column head refer to *ρ*_*tissue*_ as tissue density (kg m^-3^); *C*_*D*_*Am*^-1^ as combined drag term (m^2^ kg^-1^); *V*_*air*_ as volume of air (ml kg^-1^). Data are presented with ±95% CI in parentheses. For global parameter estimates, .g refers to the global parameter and .var refers to individual or dive-by-dive variance.

The global body tissue density was estimated with a posterior 95% credible interval (CI) of 1029.5–1033.6 kg m^-3^ (mean = 1031.6 kg m^-3^). Individual posterior mean values ranged from 1025.2 to 1043.1 with ±95% CI of 0.04–2.3 kg m^-3^. The 95% CI range for individual tissue density estimates decreased with increasing number of 5-s sub-glides in the dataset (Fig 1B). There was no significant relationship between the 95% CI range and the average depth at which the sub-glides occurred (Spearman’s rank test, p = 0.22); depth of glides ranged from 5.1 to 343.2 m with individual mean ranging 25.2 ± 10.8 to 97.3 ± 55.4 m. There was a tendency for the 95% CI ranges to be smaller for the whales tagged in Antarctica using DTAGs (0.6 ± 0.5 kg m^-3^) than in the Gulf of St Lawrence using 3MPD3GTs (2.5 ± 1.3 kg m^-3^). It is possible that different sampling frequencies and resolution of sensors as well as speed determination methods (measured/estimated) of 3MPD3GTs and DTAGs might influence the precision of tissue density estimates. Yet, the effect of the two different archival tag models could not be fully addressed due to the differences in location itself and longer data duration for the Antarctic DTAG dataset (22.9 ± 1.6 h) compared to the Gulf of St Lawrence 3MPD3GT dataset (3.5 ± 1.5 h; Table 1).

**Fig 2:**
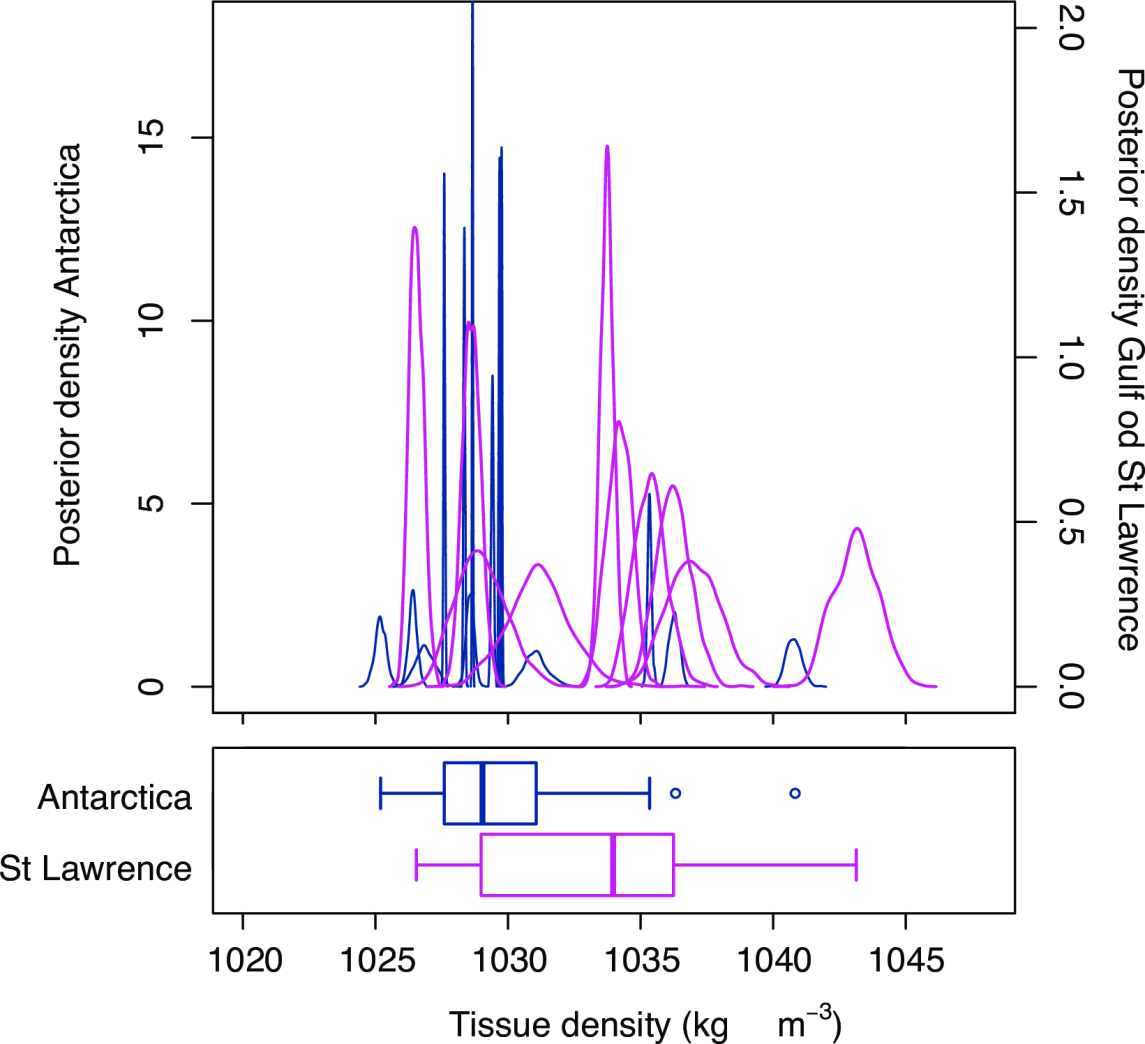
Tissue density estimates from the best model with the lowest DIC. The top panel shows posterior distribution of individual body tissue density for each tag deployment. Blue and magenta lines indicate whales from Antarctica and the Gulf of St Lawrence, respectively. Box plots in the bottom panel show median and interquartile range of tissue density estimates from each location.

**Fig 3:**
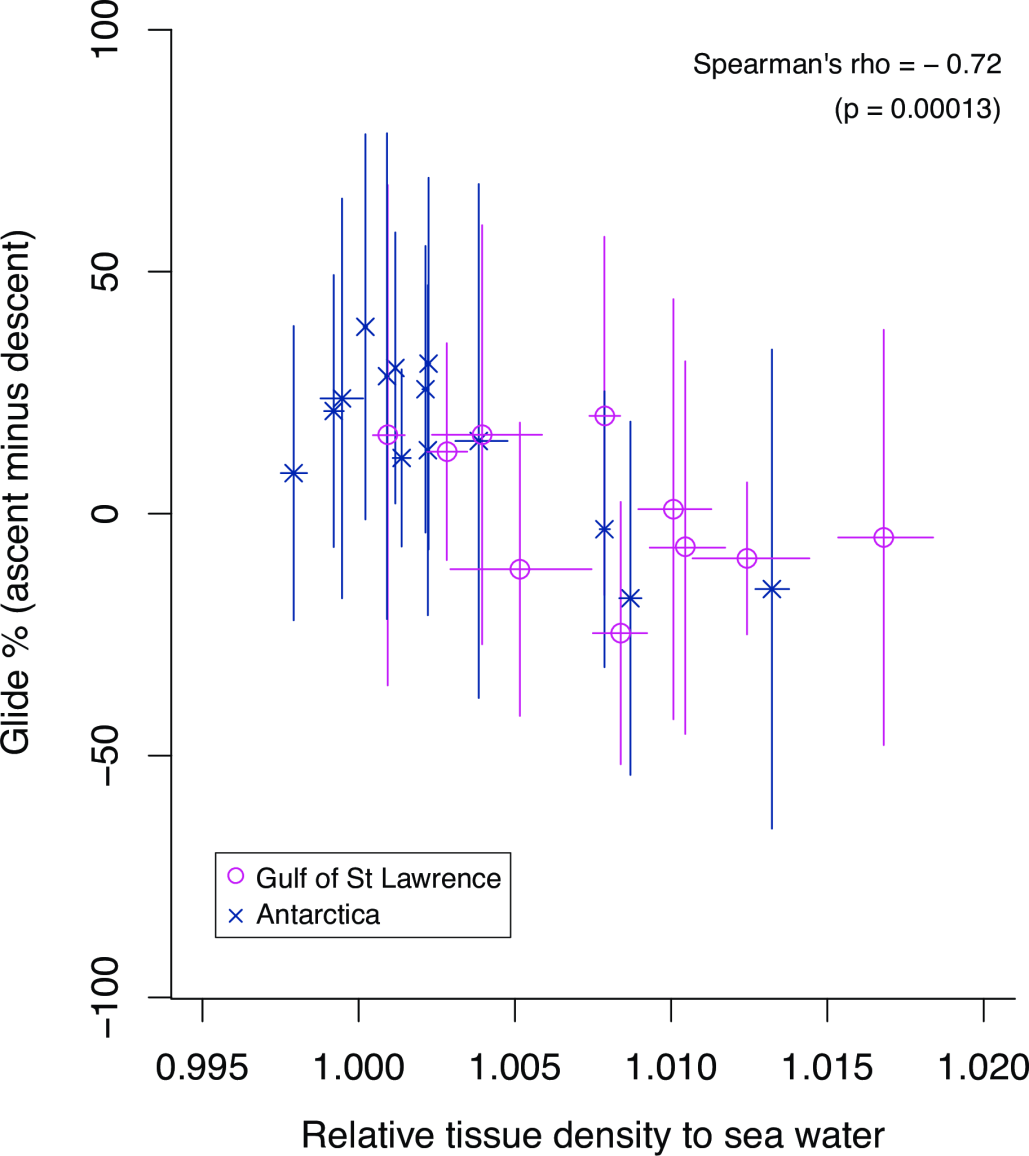
Relationship between gliding patterns and relative tissue density. The y-axis indicates differences in the percentage of time spent gliding during ascent and descent phases of non-feeding dives by each whale. Vertical and horizontal error bars show standard deviation and 95% credible interval range, respectively. A relative tissue density of >1 indicates that tissue density was denser than surrounding seawater.

Whales in the Gulf of St Lawrence had relatively higher tissue density (median = 1034.0 kg m^-3^, range = 1026.5–1043.1 kg m^-3^) than Antarctic whales (median = 1029.0 kg m^-3^, range = 1025.2–1040.8 kg m^-3^) although there was high inter-individual variation within each feeding population (Fig 2). The posterior mean tissue density of the male Mn11_H607 that was tagged twice in July and September 2011 in the Gulf of St Lawrence decreased by 5.8 kg m^-3^ in 40 days (Table 1). Tissue densities of two pregnant females were estimated as the lowest (1026.5 ± 0.5 kg m^-3^ for Mn11_H002) and the second lowest (1028.6 ± 0.7 kg m^-3^ for Mn11_H584_2) among the whales from the Gulf of St Lawrence (Table 1). There was a significant negative correlation between relative tissue density to seawater and percent time spent gliding during ascent vs decent phases of non-feeding dives (Spearman’s rho = -0.72, p <0.001; Fig 3).

**Fig 4:**
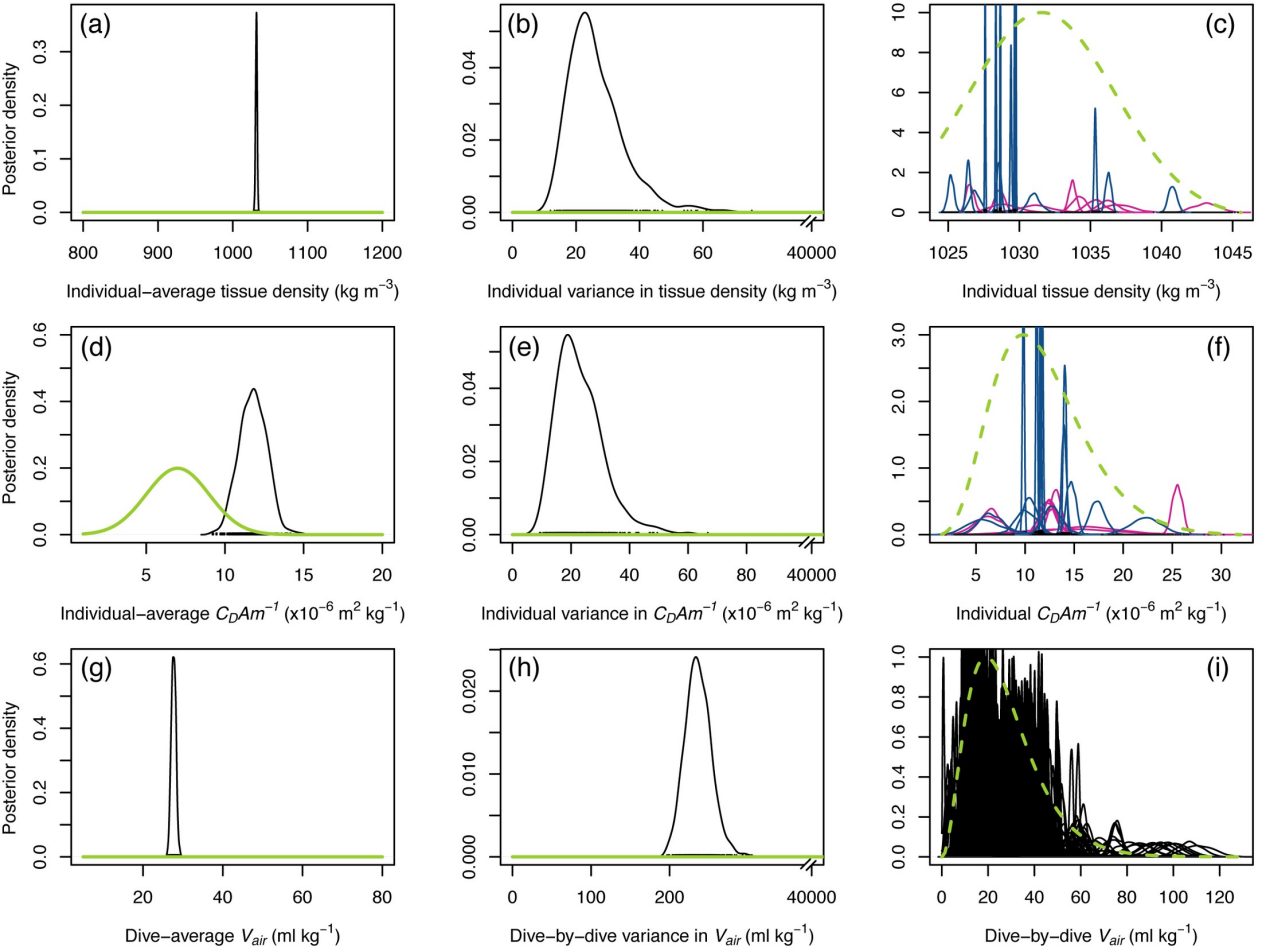
Prior and posterior distributions from the model with lowest DIC (Table 3). Prior and posterior distributions of tissue density (a, b, c), drag term (d, e, f) and diving gas volume (g, h, i) are shown in each panel. Solid green and black lines indicate the prior and posterior distributions, respectively. Dashed green lines show the estimated global distribution that can be interpreted as the population distribution for that parameter. The left and middle panels show global parameters (a, b, d, e, g, h) and the right parameters show individual and dive-specific parameters.

The posterior mean of the global drag term was 11.8 x 10^−6^ ± 1.6 x 10^−6^ m^2^ kg^-1^ (± 95% CI). The posterior mean was higher and the distribution had little overlap with the prior distribution that had a mean of 7.0 x 10^−6^ m^2^ kg^-1^ (Fig 4). The posterior means of individual drag term values ranged from 6.0 x 10^−6^ to 25.5 x 10^−6^ m^2^ kg^-1^, but most of them were near 12.5 x 10^−6^ m^2^ kg^-1^ (Table 1). The posterior mean of global diving gas volume was 27.7 ± 1.1 ml kg^-1^ (±95% CI).

The best-fitting model with the lowest DIC evaluated dive-by-dive variation in diving gas volume. The dive-by-dive estimates of diving gas volume ranged from 0.03 to 129.2 ml kg^-1^, but 90% of the estimates were within 9.2–53.5 ml kg^-1^. Diving gas volume was estimated slightly higher for feeding dives with more than 1 lunge (median = 26.3 ml kg^-1^, range = 2.6–110.8 ml kg^-1^) than other dives (median = 21.3 ml kg^-1^, range = 0.8–97.5 ml kg^-1^; Wilcoxon rank sum test, p = 0.021). Weak correlations between diving gas volume and dive depth were observed for both feeding (Spearman’s rho = 0.09, p = 0.036, N = 515) and non-feeding dives (Spearman’s rho = 0.21, p < 0.001, N = 252). However, no apparent relationships between diving gas volume and dive duration were observed for feeding (Spearman’s rho = 0.02, p = 0.58, N = 515) or non-feeding dives (Spearman’s rho = 0.05, p = 0.45, N = 252).

## Discussion

To date, the hydrodynamic glide model has been used to estimate tissue density of deep diving marine mammals such as elephant seals, sperm whales, northern bottlenose whales and long-finned pilot whales [15, 34, 38, 47]. In this study, we successfully applied this method to substantially shallower-diving humpback whales to estimate tissue density from two geographically distinct feeding populations. To examine the variability of the unknown parameters (tissue density, drag and diving gas volume), we fitted 12 models with different model structures. The best model included individual variation in tissue density and drag, supporting our expectation that each whale had different tissue density. The best-fitting model also included dive-by-dive variation in diving gas volume. Although there was no apparent overall relationship between diving gas volume and dive duration, it is possible that whales change the amount of inhaled air before dives depending on their activity [15]. The gliding patterns of whales correlated with their estimated tissue density, with denser whales spending relatively more time gliding during descent and less-dense whales spending more timing gliding during ascent phases (Fig 3). The significant correlation of tissue density and gliding patterns provides a degree of validation that the tissue density estimates, or at least their relative values, were accurate.

### Drag term estimates

The drag coefficient is one of the key parameters to estimate tissue density using the hydrodynamic glide model. Following Miller et al. [15], the combined drag term (*C*_*D*_*Am*^-1^) was estimated using a relatively narrow Gaussian prior that was determined based on auxiliary published data in order to improve the precision of tissue density estimates. However, the global (individual-average) estimate of the drag term in the best-fitting model (11.8 x 10^−6^ m^2^ kg^-1^) did not concentrate within the distribution of the prior (7.0 x 10^−6^ m^2^ kg^-1^).

As in previous studies [15, 34, 38], we neglected any specific effect of lift, although lift-related induced drag may not be negligible in the case of humpback whales due to their large pectoral flippers [48] and propensity to glide at shallow angles. It is possible that the influence of induced drag due to lift generation may explain the mismatch of the prior expectation of the combined drag term and its posterior estimate from the data. Adding the induced drag to the hydrodynamic glide model, the drag part of the equation can be expressed as

$$-0.5\rho_{sw}\frac{C_{D}A}{m}v^{2}-0.5\rho_{sw}\frac{A_{Flipper}}{\pi AR}\frac{C_{L}^{2}}{m}v^{2}$$

where *A*_*Flipper*_ is flipper surface area (m^2^), *AR* is flipper aspect ratio and *C*_*L*_ is the lift coefficient [59]. Because both the parasite drag and the induced drag are a function of speed-squared, the equation can be rewritten as

$$-0.5\rho_{sw}\left(\frac{C_{D}∙A}{m}+\frac{A_{Flipper}}{\pi ∙AR}∙\frac{C_{L}^{2}}{m}\right)\;v^{2}$$

Thus, the structure of the equation is unchanged just with the addition of induced drag to that of the parasite drag term *C*_*D*_*Am*^*-1*^.

We suggest that the model estimated higher global *C*_*D*_*Am*^-1^values due to the effect of induced drag by assuming that the model estimated the combined term in parenthesis, instead of the parasite drag term (*C*_*D*_*Am*^-1^) alone. The lift coefficient of a humpback whale flipper is estimated as 0–0.9 through wind tunnel measurements [60]. Based upon literature values for the surface area (*A*_*Flipper*_, 12.20 m^2^) and the aspect ratio (*AR*, 5.67) of a humpback whale flipper [48], *A*_*Flipper*_*C*_*L*_^2^/(π*ARm*) is estimated as 0–22 x 10^−5^ for a 12-m long whale. Adding this value to 7 x 10^−6^ (i.e. mean of the *C*_*D*_*Am*^-1^prior), the combined drag term in the parenthesis is expected to range between 7 x 10^−6^ and 29 x 10^−6^ m^2^ kg^-1^ which overlaps with the global drag term estimates in this study (11.8 x 10^−6^ ± 1.6 x 10^−6^ m^2^ kg^-1^, ± 95% CI). This suggests that the mismatch between the prior and *C*_*D*_*Am*^-1^estimates derived from the addition of the induced drag and that lift-related drag forces should not be ignored for this species.

In comparison with deeper diving marine mammals in previous studies [15, 34], the effect of lift seems particularly important for humpback whales that glide at shallower pitch angles (Table 2) where lift generation increases with correspondingly greater induced drag. Humpback whales have large flipper with a high aspect ratio that can produce lift forces to support their acrobatic movements such as high-speed turning and banking that are associated with feeding [48, 61]. In addition, the scalloped leading edge of their large flippers serves to delay stall angles and increase lift [60, 62]. Recent studies using animal-borne video camera reported that humpback whales also perform lift-generating flipper strokes for propulsion during lunge feeding [63]. In our study, we only used data during stable glides (circular variance of roll < 0.1) to minimize the influence of lift during maneuvering. However, the influence of lift-induced drag is detectable in our dataset possibly because humpback whales likely use their wing-like flippers to produce lift during stable glides at non-vertical pitch angles. Yet, it is noteworthy that our general results about tissue density seem to be robust because the model quantified the combined effect of parasite and induced drag. As a sensitivity analysis, we refitted the model using a non-informative wide range prior for the drag term instead of a narrow Gaussian prior. The resulting global average drag term was 13.1 x 10^−6^ ± 2.4 x 10^−6^ m^2^ kg^-1^ and global average tissue density was 1031.6 ± 2.1 kg m^-3^, which differed very little from the estimated values with a narrow prior. Similarly, individual tissue density estimates were nearly identical to the result of our original model, supporting the robustness of the tissue density estimates to the prior specification. Thus, the general results about tissue density seem to be robust because the model appears to have estimated a reasonable value for the combined effect of parasite and induced drag.

### Body tissue density

**Fig 5:**
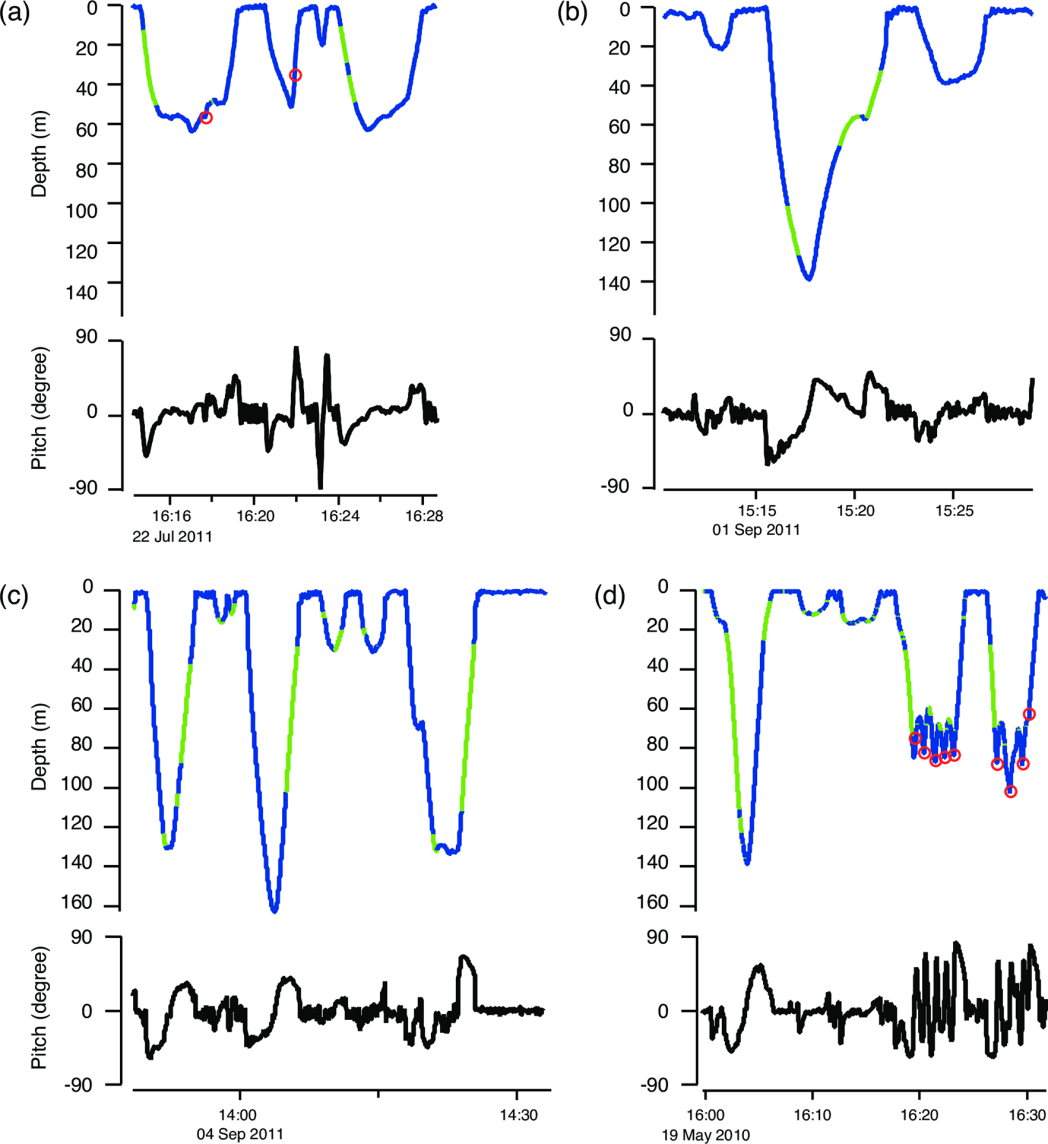
Example data records for dive profile and pitch. Dive profile with gliding and stroking periods are indicated in green and blue, respectively. Red circles indicate feeding events. Examples are taken from an adult male during (a) early feeding season (Mn11_H607_1; tissue density = 1037.0 kg m^-1^) and (b) late feeding season (Mn11_H607_2; 1031.2 kg m^-1^), (c) a pregnant female (Mn11_H002; 1026.5 kg m^-1^) and (d) a calf (Mn10_139a; 1040.8 kg m^-1^).

Estimated individual-average (global) body tissue density of humpback whales (1031.6 ± 2.1 kg m^-3^; Table 3) was similar to that of other cetaceans reported to date (1030.0 ± 0.8 kg m^-3^ for *Physeter macrocephalus* [38]; 1031.5 ± 1.0 kg m^-3^ for *Hyperoodon ampullatus* [15]), indicating that non-gas body tissues are typically denser than seawater. However, long-finned pilot whales were estimated to have even denser tissues of 1038.8 ± 1.60 kg m^-3^ [47]. For humpback whales in this study, a large variation was detected in individual-specific body tissue density ranging from 1025.2 to 1043.1 kg m^-3^, as we expected, because individual tissue density at feeding grounds would change depending on factors such as age, sex, reproductive status, prey availability and the number of days since arrival at the feeding ground [4, 6, 18, 24]. In a study of fin whales conducted using Icelandic whaling data, pregnant females had the highest rate of fattening during the feeding season as they increased their total body energy content by nearly 80% [4]. A similar trend was reported for minke whales (*Balaenoptera acutorostrata*) in Iceland: the blubber volume of pregnant females almost doubled over the feeding season [24]. Using the hydrodynamic glide model, high lipid-stores of two pregnant female humpback whales (Mn11_H002 and Mn11_H584_2) were indicated by low tissue density estimates of 1026.5 kg m^-3^ and 1028.6 kg m^-3^ that were the lowest and the second lowest, respectively, among all of the tagged whales in the Gulf of St Lawrence. A decrease in tissue density over the feeding season due to accumulation of lipid stores was also detected in this study: tissue density of a repeated sampled adult male (Mn11_H607) decreased from 1037.0 to 1031.2 kg m^-3^ in 40 days. Based on extrapolation from elephant seals, the proportion of lipid content (*P*_*lipid*_) corresponding to these tissue densities of Mn11_H607 would be 36.3% and 39.0%, as determined from *ρ*_*tissue*_ = *ρ*_*lipid*_*P*_*lipid*_+*ρ*_*lipid-free*_ (1-*P*_*lipid*_), where *ρ*_*lipid*_ and *ρ*_*lipid-free*_ are 900.7 and 1114.6 kg m^-3^, respectively [34]. Our results also showed that one of the two calves had low body tissue density of 1025.2 kg m^-3^ (Mn10_155b) in agreement with general expectation that calves are more buoyant because they deposit fat during the lactation period [64]. The other calf (Mn10_139a), however, had relatively high tissue density of 1040.8 kg m^-3^ that was supported by its gliding pattern suggestive of negative buoyancy: the whale spent more time gliding during descent (61.3%) than ascent (45.7%) phases of non-feeding dives (Fig 5D). It is possible that Mn10_139a had a poor body condition, reflecting its mother’s poor condition indicated by its relatively high body density (Mn10_139b, 1029.4 kg m^-3^) compared to the other mother in the study (Mn10_155a, 1027.6 kg m^-3^).

During the feeding season, it is essential for humpback whales to accumulate a sufficient amount of energy for survival, growth and/or reproduction. Previous studies estimated the amount of energy gained by baleen whales over the course of a feeding season via anatomical measurements and chemical analysis of multiple whale carcasses [4, 21]. More recent work has described dynamic foraging patterns of whales throughout the course of the foraging season suggesting that whales alter their feeding behaviour (rates and dive depth) commensurate with changes in the availability of prey [65]. This could lead to non-linear changes in the accumulation of energy, and, combined with body density estimates collected over similar time periods, can offer insights as to the most critical times and locations for whales to regain energy stores and how different life history classes vary. This information is critical to understanding how environmental changes and potential human disturbance can significantly impact individual and population-level health of marine mammals and other animals.

Changes in tissue density lead to changes in buoyancy that influence swimming patterns of diving animals given strong selection for them to travel efficiently to and from depth [33, 39]. For example, it is expected that animals with higher density should glide more during descent aided by negative buoyancy whereas less dense positively buoyant animals should employ more glides during ascent. In agreement with the expectation, a negative correlation between tissue density estimates and percent time spent gliding during ascent vs. descent phases of non-feeding dives was observed (Fig 3), suggesting that the model successfully detected relative differences in individual tissue density. Because there is greater variability in tissue density of humpback whales than deep diving toothed whales [15, 38, 54], the relatively low precision of tissue density estimates obtained here (±95% CI of individual tissue density ranged up to 2.3 in this study whereas to 0.4 in [15]) seem to be sufficient to detect individual and/or temporal variation. Lower precision tissue density estimates may be expected if there is high variability in acceleration that is not accounted for in the model. Therefore, sample size is particularly important to consider for the body density estimation of humpback whales for which induced drag may cause variability in gliding acceleration. The result showed that the number of 5-s sub-glides in each dataset is one of the key factors affecting the range of 95% CI for the posterior estimates of individual tissue density. Specifically for humpback whales, >200 sub-glides in each dataset seem to be needed to obtain highly precise estimates with 95% CI range of 1 kg m^-3^ (Fig 1B).

In this study, we estimated tissue density of humpback whales at two geographically distinct feeding grounds. Tissue density of whales from Antarctica and Gulf of St Lawrence largely overlapped, but there was a tendency for Antarctic whales to have lower tissue density (Fig 2), indicating that animals in that location at that time had larger lipid reserves than did the animals tagged in Canada. It is possible that the geographic differences reflected different temperature and prey conditions on two feeding grounds. However, as numerous factors can affect individual tissue density, more data, including basic information of individuals such as sex, age class and reproductive status that can be obtained from photo-ID and biopsy studies would be essential to identify factors that cause these geographic differences.

The methods used in this study closely followed methods published for other deep-diving odontocete species. Some studies used an estimated value of 0.06 for the entrained mass of water *m*_*e*_ which is moved forward along with the body of the animal [38]. Because we did not have specific measurements of animal mass in this study, we also did not include estimates for entrained mass. We also do not expect addition of a constant mass proportion of mass to all estimates would affect their relative values. However, the absolute values obtained in this study could be made more accurate with finer estimates of the length, mass and surface area of each whale as was done using photogrammetry by Miller et al. [38]. We do recommend incorporation of such data when available, and to include estimates of entrained mass to obtain accurate absolute values of body tissue density.

### Diving gas volume

The posterior mean of global (dive-average) diving gas volume was 27.7 ± 1.1 ml kg^-1^ (±95% CI). This value is substantially lower than the estimated lung volumes of mysticete fin (29–61 ml kg^-1^) and sei (*Balaenoptera borealis*) whales (116–151 ml kg^-1^), whose lung volumes were measured via inflation of excised lungs [66, 67]. This could indicate that: 1) lung volumes of mysticete whales are smaller than estimated from excised lungs in which some amount of air is likely to be trapped [46]. Piscitelli et al. [67] noted that the mass specific volume of sei whales from that study were outliers on a comparative basis relative to smaller cetaceans; 2) humpback whales in this study dove with less than their full lung capacity; or 3) our estimate was incorrect and too low.

As cetaceans appear to inhale immediately prior to diving, the diving volume of cetaceans is thought to be close to the total lung volume [68]. In fact, the calculated diving lung volume of deep-diving northern bottlenose whales (27 ml kg^-1^, [15]) was similar to the measured total lung volume of 28 ml kg^-1^ ([69] reviewed in [68]). However, shallower diving species may not always dive with full lungs; for example, the diving lung volume and the total lung volume of bottlenose dolphins are 40–50 ml kg^-1^ [70] and 50–91 ml kg^-1^, respectively (reviewed by [67]). Differences in lung sizes and thoracic morphology of shallow and deep diving cetaceans have been reported [46]. As the effect of air-derived buoyancy is stronger at shallower depth, it is possible that shallower-diving whales do not always dive with full lung capacity. A large variation in dive-by-dive estimates of diving gas volume found in this study and, albeit weak, the positive relationship between diving gas volume and dive depth would support this hypothesis. While no systematic variation of diving gas volume in relation to dive duration was detected, further detailed analysis of dive-by-dive variation in diving gas volume could provide new insights into their diving physiology.

Another possible explanation for the low estimate of diving gas volume is that the amount of gas stored in the body might decrease during dives. It has been reported that humpback whales actively exhale underwater in some situations. For example, humpback whales have a diverse repertoire of feeding behaviours, including “bubble feeding” that involves underwater exhalation to form bubble clouds, nets or curtains to corral prey [61, 71]. Bubbling is also observed in non-feeding situations such as play [71] and social interactions [72]. Although apparent bubbling was not detected from acoustic audits of the DTAG datasets in this study, it is possible that some air might passively escape from the body during dives. If such underwater exhalation and/or passive loss of air occurred, our estimate of diving gas volume would be too low because the majority of the glides used in the analysis were recorded during ascent phases of dives, and thereby the estimate reflects the amount of gas in the body at latter part of dives.

### Conclusion and future directions

We demonstrated that the hydrodynamic glide model can be used to detect individual and temporal variation in body tissue density of humpback whales, suggesting that it is likely to be broadly applicable across a range of aquatic animals including shallow diving baleen whales. The important next step is validation with other techniques such as visual assessment [28, 29], biopsy sample measurements, and photogrammetric measurements of body width versus length using overhead images [11, 30].

This study represents a cross-sectional design, in which the tissue densities of multiple animals were measured. Longitudinal tracking of changes in individuals’ tissue density as has been done with elephant seals drift dives [5], or repeated measurements as we made for whale Mn11_H607, may be a more powerful approach to determine specific factors that affect the lipid-store body condition of humpback whales. Considering that humpback whales are less difficult to tag such that multiple tagging of the same individual is possible, this tag-based minimally invasive approach may provide an effective tool to monitor body tissue density as a measure of body condition. By integrating life-history data of individuals (e.g. age, sex, size, reproductive status) as well as prey availability at feeding grounds, this approach can be helpful to understand bioenergetics and health of individual whales within increasingly human-altered ecosystems. Ultimately, tracking the tissue density of individual whales using longer duration tags could be a powerful technique to relate their body condition to how they interact with features of their natural environment.

## Supporting information

S1 Fig Examples of linear regression of speed over time to estimate acceleration during 5s sub-glides. Linear regression of speed over time was conducted at each 5s sub-glide to estimate acceleration as a slope of the regression line. Top and bottom panels show examples from 3MPD3GT and DTAG deployments, respectively.
(TIF)

S1 File Data of all 5-s sub glides performed by tagged humpback whales. This file includes all 5-s sub glides data used in this study.
(CSV)
