# Supplementary figures and images for: Body density of humpback whales (Megaptera novaengliae) in feeding aggregations estimated from hydrodynamic gliding performance
Source: PLoS One. 2018 Jul 12;13(7):e0200287. doi: 10.1371/journal.pone.0200287 (PMC6042725; doi:10.1371/journal.pone.0200287)

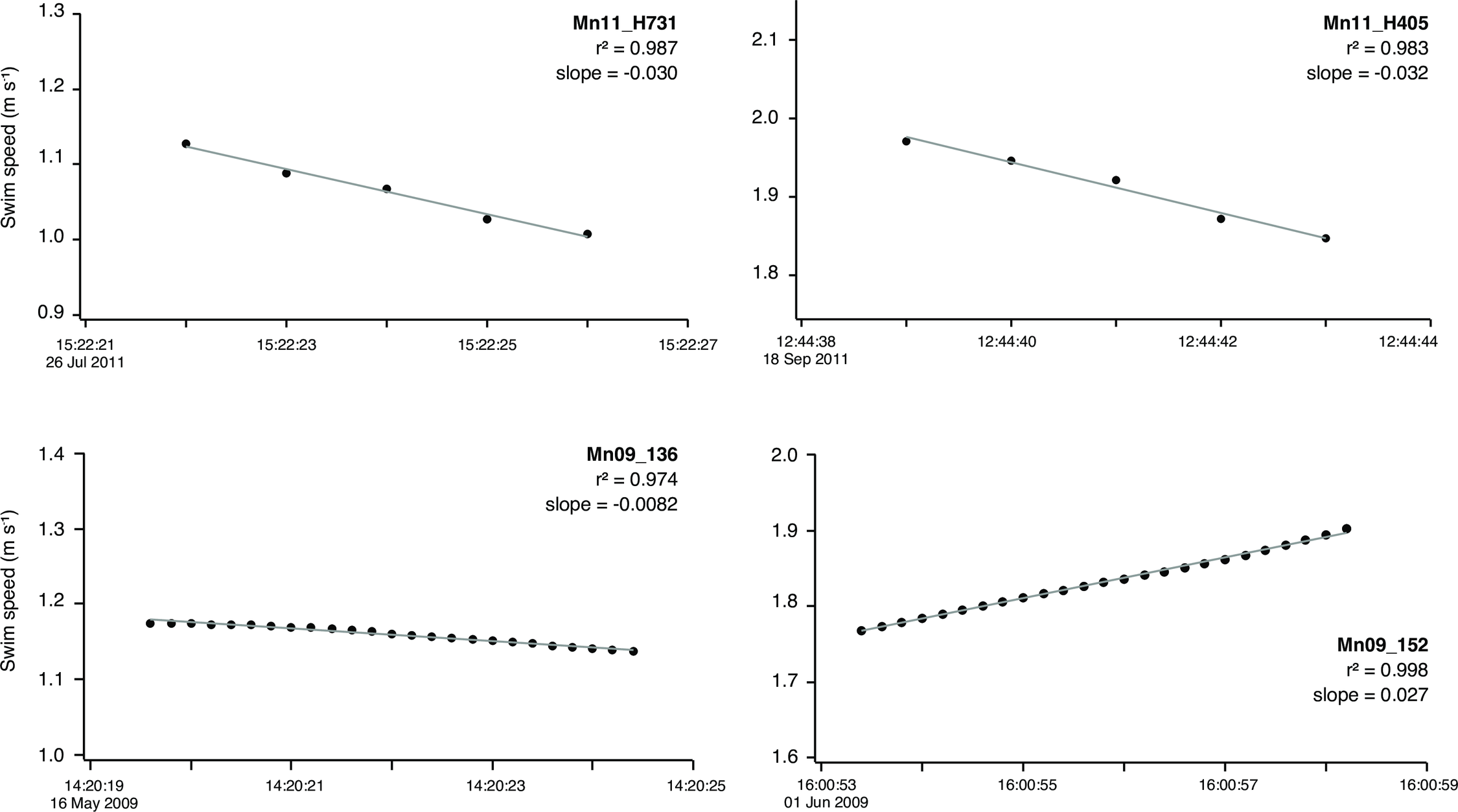

Supplement: S1 Fig — Linear regression of speed over time was conducted at each 5s sub-glide to estimate acceleration as a slope of the regression line. Top and bottom panels show examples from 3MPD3GT and DTAG deployments, respectively. (TIF) [file pone.0200287.s001.tif]
